# Long-term management of recurrent uveitis associated with autoimmune lymphoproliferative syndrome

**DOI:** 10.1186/s12348-025-00530-5

**Published:** 2025-09-24

**Authors:** Timothy Kaftan, Nam V. Nguyen, Jack Begley, Helen Song, Brent Timperley, Eric Suhler, Thomas Hejkal, H. Nida Sen, Marcus Snow, Steven Yeh

**Affiliations:** 1 Department of Ophthalmology, Stanley M. Truhlsen Eye Institute, University of Nebraska Medical Center, Omaha, NE USA; 2 College of Medicine, University of Nebraska Medical Center, Omaha, NE USA; 3 Department of Ophthalmology, Casey Eye Institute, Oregon Health and Science University, Portland, OR USA; 4 Eye Consultants, P.C, Omaha, NE USA; 5 Department of Ophthalmology, George Washington University School of Medicine & Health Sciences, Washington, DC USA; 6 Department of Internal Medicine, Division of Rheumatology, University of Nebraska Medical Center, Omaha, NE USA; 7 Emory Eye Center, Emory University School of Medicine, Atlanta, GA USA

**Keywords:** Autoimmune lymphoproliferative syndrome, Anterior uveitis, Immunosuppression, Methotrexate, Topical corticosteroid

## Abstract

**Background:**

The purpose of this case report is to describe the long-term findings and management of recurrent uveitis in a patient with autoimmune lymphoproliferative syndrome (ALPS).

**Case observation:**

A 25-year-old female with a history of ALPS and previous uveitis flares presented with reduced vision and pain in the left eye. Uveitis flares began at age 4 and continued into adulthood prompting multiple medical regimen changes. Ocular involvement included anterior uveitis, posterior uveitis, and panuveitis. The laterality of uveitis flares favored the left eye, however bilateral episodes were documented. The patient was diagnosed with recurrent anterior uveitis and treated with topical corticosteroids with subsequent resolution of her symptoms. However, the patient continued to develop recurrent uveitis prompting low-dose long-term maintenance treatment with topical corticosteroids. Additionally, methotrexate was added which led to uveitis resolution and inactive disease for over a year on this treatment regimen.

**Conclusions:**

Uveitis secondary to chronic autoimmune disease, such as ALPS, offer long-term challenges for ocular health management. Due to the recurrent nature of uveitis flares in patients with ALPS, long-term ophthalmic and systemic management may be warranted with low-dose maintenance topical corticosteroid or systemic immunomodulation. Response to topical and systemic therapy can be highly patient specific. Complex side effect profiles of many systemic immunotherapies make drug tolerability an important treatment consideration. Multidisciplinary coordination of systemic immunosuppression should be tailored to the patient’s anti-inflammatory needs, as well as careful monitoring of adverse events.

## Background

Autoimmune lymphoproliferative syndrome (ALPS) is an inherited lymphoproliferative disorder caused by mutations in the genes encoding for FAS pathway proteins and receptors [1]. Mutations are primarily inherited in an autosomal dominant fashion, although rare, biallelic mutations have been identified in autosomal recessive inheritance patterns [1]. The FAS pathway is responsible for inducing apoptosis to eliminate defective or self-targeting immune cell populations [1, 2]. Mutations in this pathway, as seen in ALPS, can result in unregulated proliferation of mature, autoreactive T lymphocytes, which may manifest as autoimmunity affecting multiple organ systems, including the eye. ALPS typically presents in early childhood; however, due to the rare nature of the disease, many patients are not diagnosed until years after symptoms develop [3]. The condition is commonly characterized by cytopenia, hypogammaglobulinemia, splenomegaly, and hepatomegaly [3]. Due to the defective T cell function, patients with ALPS are at increased risk for both infection and lymphoma [3, 4]. Severe autoimmune syndromes associated with ALPS, including glomerulonephritis, autoimmune hepatitis, and uveitis, have been reported in the literature [1, 3].

Cases of ALPS-associated uveitis have been reported infrequently in the literature, and the long-term disease management has been scarcely described [5]. The purpose of this case report is to describe the ocular and systemic manifestations of a patient with ALPS whose initial manifestations and early treatment course were previously reported [5], now with findings in adulthood. This report highlights the challenges and importance of long-term uveitis management with multidisciplinary care for patients with ALPS.

## Case observation

### ALPS presentation, diagnosis, and management

**Fig. 1:**
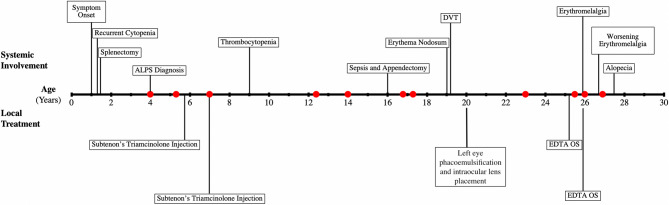
Timeline of major systemic manifestations and ocular treatment of autoimmune lymphoproliferative syndrome. Complications listed chronologically with non-ocular complications above the horizontal axis, and ocular targeted therapies below the horizontal axis. The patient’s uveitis episodes are indicated by a red circle on the horizontal axis of the timeline.

A 15-month-old female was hospitalized for febrile neutropenia with hepatosplenomegaly, and following extensive work-up, she was presumptively diagnosed with idiopathic thrombocytopenic purpura [5]. Following the diagnosis, she was treated with IVIG, prednisone therapy, and eventually, she underwent splenectomy. In the following years, she continued to develop episodes of cytopenia and pneumococcal bacteremia prompting a reevaluation of diagnosis at age 4. At that time, laboratory and genetic studies revealed classical ALPS findings of increased TCR αβ^+^CD4^−^CD8^−^ T cells and FAS gene mutation. Subsequently, she was given cyclosporine for systemic immunosuppression. At the same time as her ALPS diagnosis, she was diagnosed with bilateral uveitis, which was managed with topical corticosteroids. Over the course of her disease management, the patient received multiple regimens of systemic immunosuppressive therapy including oral prednisone, cyclosporine, mycophenolate mofetil, tacrolimus, and sirolimus for immune dysregulation associated with ALPS. Suboptimal anti-inflammatory response and intolerance of side effects were observed with the various therapies, ultimately leading to continued modification of her treatment regimen. The chronology of systemic and ocular complications is summarized in Fig. 1.

Due to the immune dysregulation secondary to ALPS, history of splenectomy, and the need for long-term immunosuppressive treatment, the patient was placed on daily penicillin therapy. Despite the antibiotic maintenance therapy, she had multiple complications related to ALPS and her immunocompromised state. These complications included pelvic inflammatory disease, appendicitis requiring appendectomy, and subsequent sepsis and erythromelalgia. While worth noting the association between cyclosporine use [6] and erythromelalgia, given her lack of symptoms with prior cyclosporine use, the erythromelalgia was deemed secondary to the underlying ALPS. After an unsuccessful trial of gabapentin, symptom relief coincided with initiation of weekly methotrexate, introduced primarily for its uveitis management.

### Ocular involvement of ALPS and initial management

Following the patient’s diagnosis of bilateral uveitis episode at age 4, she continued to follow up with her local ophthalmologist and the uveitis service at the National Eye Institute [5]. Her uveitis recurred at age 5, and this episode did not respond immediately to topical prednisolone warranting further treatment with periocular triamcinolone injection. Inflammatory quiescence was achieved over the next month with the addition of a topical rimexolone taper. At age 7, she again experienced a uveitis flare and was treated with a periocular triamcinolone injection along with a topical prednisolone taper. At this time, it was determined that the patient would benefit from long-term local anti-inflammatory therapy in the form of prednisolone drops twice daily. After two years without ocular or systemic autoimmune flares, this regimen was discontinued.

She developed uveitis recurrence at age 12 and was managed with a topical prednisolone taper. Following uveitis recurrence at age 14, prophylactic prednisolone drops were continued until her next flare at 16 years old. Notably, this was her first uveitis recurrence while receiving maintenance-dosing topical prednisolone. Topical corticosteroid was then escalated to difluprednate for acute uveitis management. Following initial resolution, uveitis flare-ups prompted difluprednate at ages 17 and 19. At age 19, oral tacrolimus was added to achieve uveitis quiescence but was stopped due to systemic side effects. Additionally, the patient required cataract surgery of the left eye at age 20 likely due to a combination of chronic topical steroid use and recurrent ocular inflammation. The patient’s next uveitis flare-up at age 23 responded promptly to a course of difluprednate taper. Notably, at age 25, several months prior to acute presentation to our clinic, the patient required ethylenediaminetetraacetic acid (EDTA) chelation in the left eye, likely due to band keratopathy. 

### Acute presentation and management

**Fig. 2:**
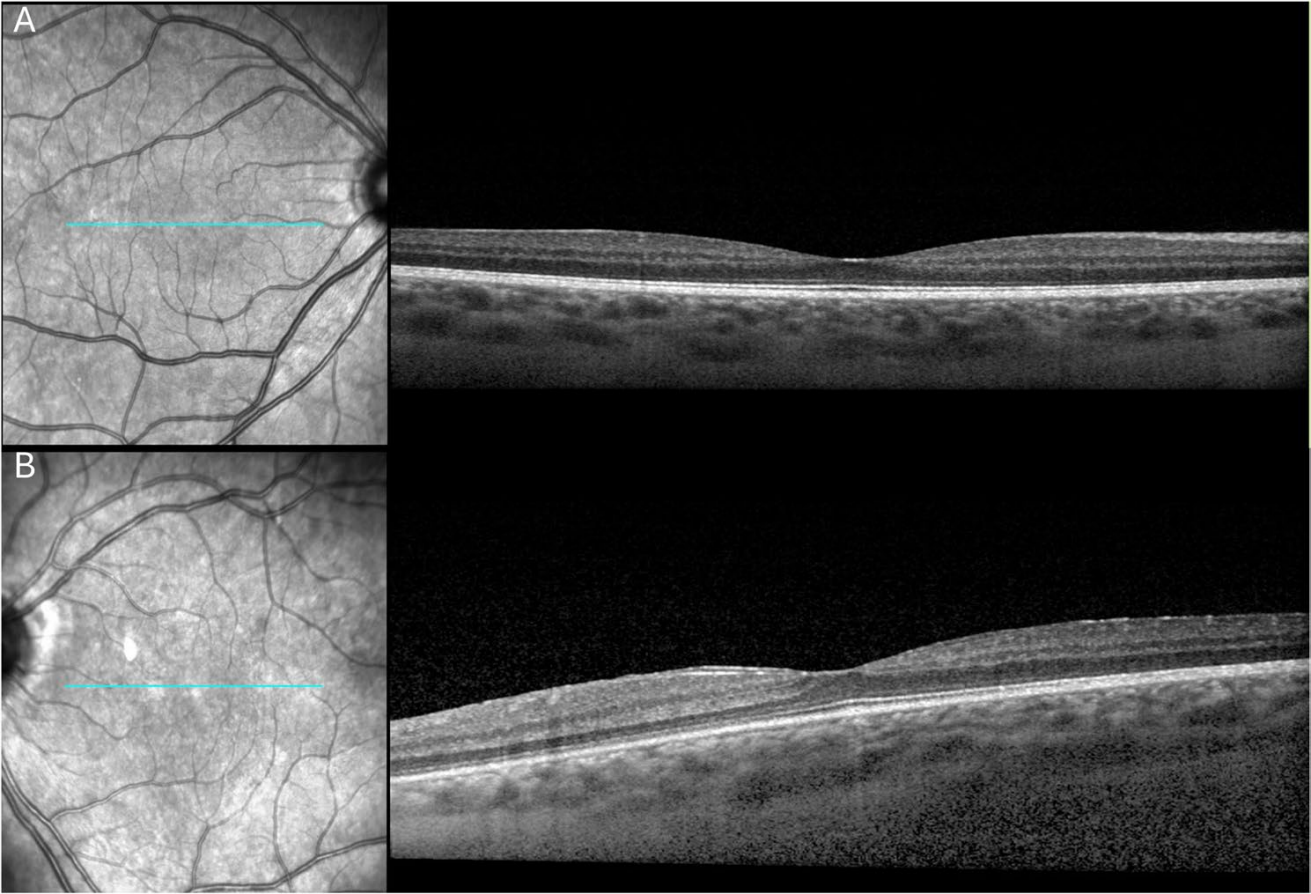
Optical coherence tomography of the right (OD) **A** and left (OS) **B** eyes at the initial visit at our clinic. The right eye demonstrated normal foveal contour without evidence of intraretinal or subretinal fluid, and the left eye showed mild epiretinal membrane without evidence of intraretinal or subretinal fluid.

**Fig. 3:**
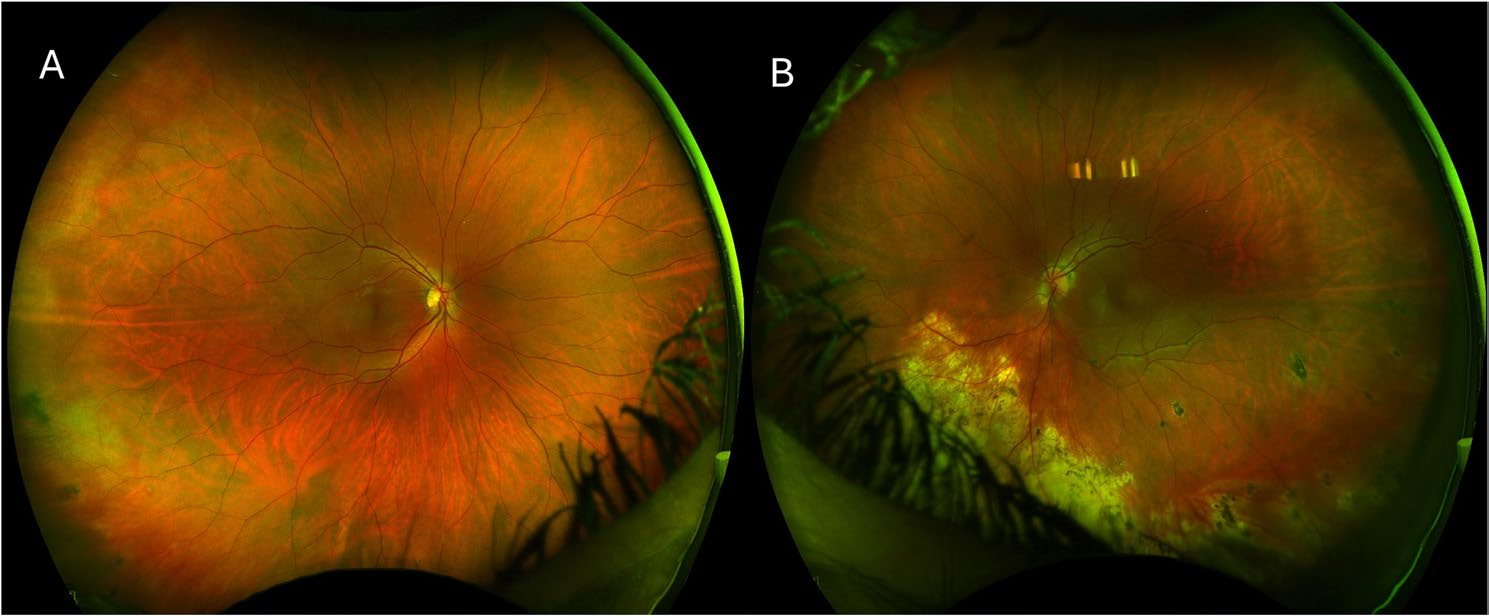
Ultra-widefield fundus photos revealed normal fundus OD **A** and significant chorioretinal scars in the inferior hemiretina OS **B.**

At age 25 years, the patient presented to our service with acute onset symptoms of eye pain, redness, and blurred vision in the left eye (OS). Best-corrected visual acuity (BCVA) was 20/20 in the right eye (OD) and 20/30 OS. Intraocular pressure was within normal limits. Slit lamp examination revealed trace anterior chamber (AC) cells OD and 2 + flare and 1 + AC cells OS. Fundus examination was unremarkable OD and showed significant chorioretinal scarring without signs of active posterior uveitis OS. Optical coherence tomography (OCT) was unremarkable OD and demonstrated mild epiretinal membrane without macular edema OS (Fig. 2). The patient was diagnosed with anterior uveitis and started on a taper of difluprednate tapered over four weeks. On 1-month follow-up, BCVA improved to 20/25 OS. Slit-lamp examination revealed quiet AC OD and minimal flare without cells OS. Fundus examination demonstrated stable chorioretinal scarring OS (Fig. 3). The patient was started on long-term maintenance therapy of difluprednate once every other day in the left eye.

Six months after the initial visit at our clinic, the patient returned to clinic with painless vision loss in the left eye. The BCVA was 20/40 OS, and the patient’s slit lamp examination was significant for trace conjunctival hyperemia and peripheral band keratopathy. The decreased visual acuity was attributed to worsening band keratopathy and dry eye syndrome. Following supportive care with artificial tears and lubrication, the patient underwent repeat EDTA chelation and was started on artificial tears and was provided with a bandage contact lens. Following chelation, BCVA improved to 20/20 OS.

One year later, at age 27, the patient’s uveitis recurred in the left eye with both AC inflammation and vitreous haze with a reduction of VA to 20/600 OS. Examination demonstrated mild anterior chamber inflammation, posterior capsule opacification, and anterior lens surface deposits consistent with recurrent anterior uveitis. Difluprednate and artificial tears were increased to five times daily, and four times daily, respectively. Follow-up examination one week later showed persistent anterior uveitis and BCVA of count fingers at 3 feet OS. Difluprednate was increased to six times per day. Additionally, the patient was started on a combination ointment with neomycin, polymyxin B, and dexamethasone (Maxitrol) nightly and homatropine daily. Difluprednate taper was extended over a period of three months and methotrexate 12.5 mg/weekly was started for uveitis symptoms. The patient responded well with an extended taper with no evidence of recurrent uveitis.

### Response to daily topical corticosteroids and Long-term uveitis Follow-up

The patient has not had uveitis recurrence for over one year on a regimen of 12.5 mg of methotrexate weekly with folic acid 1 mg daily. OCT findings at the most recent follow-up visit were stable in both eyes, and clinical exam showed no evidence of inflammation. Her most recent BCVA in the left eye has remained stable at 20/30.

## Discussion

In this case report, we have highlighted the disease course and the challenges in management of a rare case of recurrent uveitis in the setting of ALPS. The exact incidence of uveitis in ALPS is unknown due to its rare nature and difficulty in diagnosis; however, estimates have shown that uveitis occurs in around 2% of ALPS patients [7]. A broad spectrum of anatomic locations including anterior [5, 7], intermediate [7], posterior [7], and panuveitis [7, 8] have been described in association with ALPS. These findings suggest that patients with ALPS require long-term follow-up and evaluation, particularly in the context of new visual symptoms, which may herald recurrent inflammation.

Systemic immunosuppression may be helpful in the prevention of vision loss and inflammatory complications in patients with ALPS with various regimens proposed in the literature [3, 5, 7, 8]. Initial treatments proposed for patients with ALPS have included corticosteroids [3]and IVIG [3, 7]. Corticosteroid (CS)-sparing immunosuppressants such as sirolimus [3]rapamycin [8]or mycophenolate mofetil [5, 7] have been used when inflammatory resolution is not achieved following initial therapy and monitoring for adverse events is required [3, 5, 7, 8]. Additionally, biologics such as rituximab [5, 7] have been added as a third-line therapy. Our patient required multiple regimen changes due to adverse events and required a tailored approach with multi-disciplinary care. Given the breadth of medical records and use of multiple healthcare recording systems, the exact systemic regimens and corresponding dates were at-times ambiguous. However, it was determined that uveitis flares at ages 14, 16, 17, and 25 years occurred despite ongoing systemic immunosuppression with cyclosporine and mycophenolate mofetil. As with our patient, methotrexate has shown potential effectiveness and tolerability as a corticosteroid-sparing agent in the management of non-infectious uveitis [9]. Notably, methotrexate was trialed once before in the patient’s history following uveitis flare-up at age 5. The exact date and indication of methotrexate discontinuation was not explicitly found in the patient’s chart; however, the patient was not taking methotrexate during her next chronological uveitis flare at age 7. While the patient previously demonstrated a long window of inactive ocular disease, her history of uveitis flares refractory to other ongoing immunosuppressants and favorable side effect toleration supported the continued use of methotrexate for systemic immunosuppression.

Complete uveitis remission in ALPS patients has been rarely described in the literature following hematopoietic stem cell transplant [7]but is not commonly used because of the risk of severe recalcitrant cytopenia and increased risk of sepsis. Uveitis flares often require topical corticosteroid drops in addition to systemic steroid-sparing immunosuppressant agents. While long-term use of topical corticosteroid eye drops for the management and prevention of recurrent uveitis is generally not advised, disease severity should be weighed against potential adverse effects associated with other local and systemic treatment options [9]. 

In addition to long-term systemic immunosuppression, topical anti-inflammatory therapy to prevent flare-ups may be considered in patients with severe recurrent uveitis secondary to ALPS. Our patient was previously managed with intermittent tapers of prednisolone drops following uveitis recurrence. Due to the well-established adverse effects of chronic topical corticosteroid (i.e. cataract development or intraocular hypertension [10, 11]), patients are often prescribed the lowest dose necessary to achieve immune suppression [9]. Thus far, our patient’s intraocular pressure (IOP) has been within normal range, and she has not shown any visual defects suggestive of increased IOP. However, steroid use likely contributed to worsening of her posterior subcapsular cataract, first diagnosed at age 5, which required cataract extraction at age 20. Our patient’s cataract progression highlights a potential adverse effect of prolonged steroid exposure.

As an alternative to prednisolone, difluprednate is a derivative of prednisolone and has been shown to have increased absorption, penetration, and potency compared to prednisolone [10–12]. Difluprednate has also been shown to have a similar safety profile to prednisolone [11, 12]. The risk of elevated intraocular pressure with difluprednate may be mitigated with the avoidance of concurrent systemic corticosteroids, but close monitoring is needed [12]. Our patient’s success with long-term corticosteroid drops with alternate day dosing and judicious dosage reduction may indicate a role for its use in the management of uveitis with episodic recurrences secondary to ALPS.

## Conclusion

Recurrent uveitis secondary to ALPS requires ongoing immunosuppression and multidisciplinary care coordination. The case described above highlights the potential need for long-term local anti-inflammatory therapy with systemic immunosuppressive therapy. Due to the likelihood of uveitis recurrence, monitoring is advised in ALPS patients with prompt evaluation for symptom recurrence.

## Data Availability

No datasets were generated or analysed during the current study.

## Abbreviations

ALPS: Autoimmune lymphoproliferative syndrome
OS: Oculus sinister (left eye)
OD: Oculus dexter (right eye)
BCVA: Best-corrected visual acuity
AC: Anterior chamber
OCT: Optical coherence tomography
CS: Corticosteroids
